# Modular and Versatile Trans‐Encoded Genetic Switches

**DOI:** 10.1002/anie.202001372

**Published:** 2020-07-27

**Authors:** Avishek Paul, Eliza M. Warszawik, Mark Loznik, Arnold J. Boersma, Andreas Herrmann

**Affiliations:** ^1^ Zernike Institute for Advanced Materials University of Groningen Nijenborgh 4 9747 AG Groningen The Netherlands; ^2^ DWI-Leibniz Institute for Interactive Materials Forckenbeckstr. 50 52056 Aachen Germany; ^3^ Institute of Technical and Macromolecular Chemistry RWTH Aachen University Worringerweg 2 52074 Aachen Germany

**Keywords:** RNA, RNA logic gates, RNA switches, synthetic biology, tRNA

## Abstract

Current bacterial RNA switches suffer from lack of versatile inputs and are difficult to engineer. We present versatile and modular RNA switches that are trans‐encoded and based on tRNA‐mimicking structures (TMSs). These switches provide a high degree of freedom for reengineering and can thus be designed to accept a wide range of inputs, including RNA, small molecules, and proteins. This powerful approach enables control of the translation of protein expression from plasmid and genome DNA.

## Graphical abstract

**Switch‐hitter**: A bacterial genetic switch that accepts RNAs, small molecules, and proteins as inputs was developed based on a tRNA‐mimicking structure. The switch is trans‐encoded, which makes manipulation of the target mRNA redundant. It is stable to RNase, shows a strong dynamic response, and exhibits a high level of orthogonality.

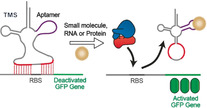

Synthetic non‐coding RNAs with simple and limited conformational states have been deployed to build RNA switches to control bacterial translation.[^1^, ^2^, ^3^] Many RNA switches suffer from low dynamic response and lack of versatility with respect to input signals that can be processed. Nonetheless, great progress involving sophisticated RNA switches has been made.[^4^, ^5^, ^6^, ^7^, ^8^, ^9^, ^10^, ^11^] These RNA switches act in cis on the target mRNA (i.e., act on the same molecule, while trans‐acting switches act on a different molecule) to achieve fast and efficient functionality with high on/off‐ratio. The “toehold switch” is a prime example of such an RNA device with strong dynamic response, orthogonality, and possibility for logic‐gate operations.^[12]^ There are also ligand‐dependent riboswitches that are cis‐acting with regard to the target mRNA.^[13]^ The incorporation of cis‐encoded switches at the leader mRNA may however interfere with folding of downstream mRNA^[14]^ and will require genetic modification upstream of the target gene. The importance of the leader mRNA sequence has been well documented for 5′‐UTR‐encoded regulators of pathogenic bacteria.^[15]^ Trans‐encoded switches would not present sequence constraints on the target mRNA. For example, a trans‐encoded switch based on the looped antisense oligonucleotide (LASO) can repress a downstream gene in presence of an input RNA^[16]^ but does not accept other signals such as small molecules or proteins. Hence, limited by their design, current artificial switches lack modularity with respect to input signals.

To overcome these limitations, we present trans‐encoded genetic switches based on a tRNA‐mimicking structure (TMS; Figure 1). Bacterial tRNA is stable to RNase and allows stable expression of RNA constructs in the cells.[^17^, ^18^] Since the TMS switch is trans‐encoded, it does not disturb the secondary structure of the target mRNA. The concept of TMS is based on metazoan mitochondrial tRNAs that display diverse sequences and structures.^[19]^ We hypothesized that sequence variation of different arms of the tRNA structure would allow easy incorporation of modules with different functions into the switch.

**Figure 1:**
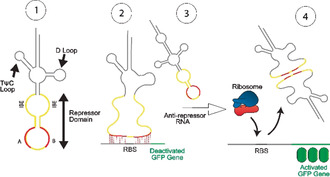
Concept to switch protein translation: Modification of the anticodon loop of a tRNA (1) blocks ribosome binding (2), which can be reversed by an anti‐repressor RNA (3), thereby allowing GFP expression (4). A and B denote the two subdomains in the repressor domain. The initial binding element (IBE) hybridizes with the antirepressor RNA.

To achieve tight control over gene expression, we designed the switch to bind both flanking sites of the ribosome binding site (RBS) of the target mRNA, without disturbing the RBS and the start codon, to block ribosome entry and subsequent translation.

We incorporated a repressor domain into the anticodon loop of the bacterial tRNA^lys^ that binds flanking sites of the RBS (Figure 1 and Figure S1 in the Supporting Information). To reverse the effect of the repressor, we designed an anti‐repressor RNA that binds the TMS through loop–loop interactions and pulls off the repressor domain from the mRNA, thereby liberating the RBS for binding to the ribosome. To provide initiation sites for the binding between the TMS switch and the anti‐repressor RNA, we incorporated two 9 nt initial binding elements (IBEs), one at each end of the repressor domain of the TMS switch. To make the design process simple, we consider the IBEs as a part of the repressor domain. We name this switch d‐TMS^IBE^.

For characterization of the switch, we used a two‐plasmid system^[11]^ and co‐expressed the anticodon‐modified TMS switch and a GFP reporter from the two separate plasmids (Figure S2) in *Escherichia coli* BL21(DE3) cells. We varied the length of regions A and B in the repressor domain and tested their ability to repress GFP by flow cytometry. Three out of eight d‐TMS^IBE^ switches showed effective GFP repression (Figure S3). These three switches contain the longest A and B domains (>12 nts), likely because the 8 nt transmitter domain needs to dehybridize for the switch to bind the mRNA. We selected the d‐TMS^IBE^ switch with the longest regions of A=10 nt and B=8 nt to obtain the best binding characteristics. We determined the minimum length of the stem in the repressor domain and the stem that connects the repressor domain with the tRNA structure (Figure S4). The need for a minimum stem length is likely due to the stability provided by a tRNA structure in an RNAse environment (see below). The d‐TMS^IBE^ switch maintains its functionality even with a variable loop of 1 nt length.

Useful switches need to be stable to degradation by RNAses. For example, a trans‐encoded bacterial switch that binds the protein Hfq is stable to RNAse.^[20]^ To investigate the stability of the d‐TMS^IBE^ switch in RNase environment, we compared repression of GFP expression by the d‐TMS^IBE^ switch with an RNA oligomer containing only the repressor sequence (Figure S5). The d‐TMS^IBE^ switch repressed GFP expression 21‐fold more effectively than the RNA oligomer, thus demonstrating that the d‐TMS^IBE^ provides stability to the RNA‐based switch in an environment where RNAse is present.

To reverse the repression of the GFP gene by the d‐TMS^IBE^ switch, we simultaneously expressed an anti‐repressor RNA from the d‐TMS^IBE^ switch plasmid (Figure S6). We again used a TMS structure for the anti‐repressor RNA to provide stability. Cells expressing both the anti‐repressor RNA and the d‐TMS^IBE^ switch showed almost the same GFP intensity as in absence of both interacting TMSs (Figure 2 a). The ON/OFF ratio of the d‐TMS^IBE^ switch was around 200‐fold.

**Figure 2:**
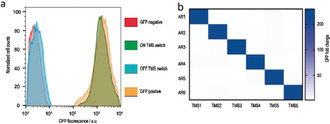
a) GFP fluorescence measured by flow cytometry for the anticodon‐modified TMS switch in the ON and OFF states (presence and absence of the anti‐repressor RNA). Negative control is without GFP induction, positive control is with GFP induction in the absence of TMS. b) Orthogonality of six different d‐TMS^IBE^ switches and their cognate anti‐repressor RNAs. The GFP fold change is the GFP fluorescence of the d‐TMS^IBE^ switch ON divided by the OFF state without background correction.

Next, we determined the orthogonality of six d‐TMS^IBE^ switches and their cognate anti‐repressor RNAs (Figure 2 b). We varied the repressor domain sequence of the d‐TMS^IBE^ switches while retaining the ability to bind to the target mRNA. Combination of each of the d‐TMS^IBE^ switches with each of the anti‐repressor RNAs resulted in fold changes in GFP expression varying between 180 and 210 for each cognate pair and less than 2 for each non‐cognate pair. Hence, our system is highly selective concerning the d‐TMS^IBE^ switch and cognate anti‐repressor RNA.

To increase the versatility of input signals, we inserted an aptamer as an additional module into the d‐loop of the TMS switch. The d‐loop determines the tertiary structure of tRNA. Previously, an aptamer was coupled to a self‐cleaving ribozyme in order to create a ligand‐dependent riboswitch,[^21^, ^22^, ^23^] which was stabilized at its base‐paired stem by binding its ligand. In our case, we hypothesize that such stabilization of a base‐paired stem in the d‐loop would provide the energy to regain the tRNA structure with concomitant release of the TMS from the mRNA. The well‐studied hybridized structure of the neomycin B (NeoB) aptamer shows that it has increased stability upon neomycin B binding.[^24^, ^25^] To verify the TMS switch activity against a small molecule, we selected azide‐modified neomycin B^[26]^ as input.

We replaced the d‐loop of the TMS switch with a neomycin B aptamer^[27]^ sequence (NeoB‐TMS^‐IBE^, Figure 3 a and Figure S7). The NeoB aptamer exhibits a low micromolar binding affinity to azide‐modified neomycin B (Figure S8). Modifying the 2‐deoxystreptamine ring of neomycin B with an azide reduces its antibacterial activity (Figure S9 and S10) and allows its use as a non‐bioactive input signal at lower concentrations. We removed the IBE elements at the repressor domain. With the incorporation of the neomycin B aptamer, the TMS switch is now comprised of a sensor (that recognizes a small‐molecule input signal), an actuator (that controls the output signal), and a transmitter (that channels the signal from the sensor to the actuator; see Figure 3 a top left). This RNA architecture responds in a concentration‐dependent manner to azide‐modified neomycin B as input (Figure 3 b), thereby demonstrating that the NeoB‐TMS^‐IBE^ structure can function as an analogue genetic switch against a small‐molecule input signal.^[28]^

**Figure 3:**
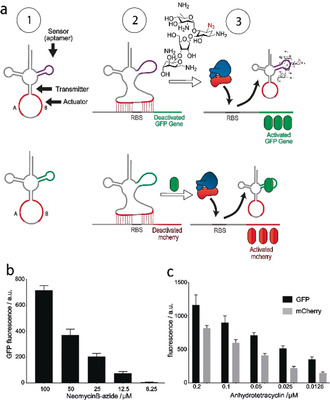
a) Controlling gene expression by the NeoB‐TMS^‐IBE^ and GFP‐TMS^‐IBE^ switches (1). Binding of the switches prevents ribosome binding (2), which is reversed by binding of the corresponding aptamer ligand (3). The GFP‐TMS^‐IBE^ switch controls mCherry expression. b) Titration of azide‐conjugated neomycin B leads to increased GFP production. c) Inducing GFP expression with anhydrotetracyclin leads to increasing mCherry fluorescence.

In addition to small molecules, the switch accepts GFP as an input signal with a GFP aptamer^[29]^ integrated into the d‐loop of the TMS (GFP‐TMS^‐IBE^, Figure 3 a). We used mCherry as an output signal, controlled under an arabinose promoter, while inducing GFP with anhydrotetracycline. The behavior of the GFP‐TMS^‐IBE^ switch against a gradient GFP input signal was similar as that of the NeoB‐TMS^‐IBE^ switch with the small‐molecule input signal (Figure 3 c). To demonstrate that the RNA switch can function as an OR logic gate in live cells, we programmed this gate by incorporating a repressor domain and the neomycin B aptamer together in a single TMS switch (NeoB‐TMS^+IBE^, Figure 4). The logic gate gave the expected output signal: Cells only express GFP in the presence of the anti‐repressor RNA or azide‐modified neomycin B (Figure 4).

**Figure 4:**
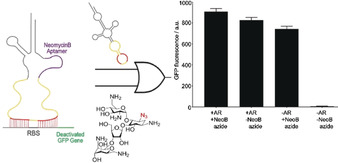
Design of an OR logic gate with the NeoB‐TMS^IBE^ switch, responding to its cognate anti‐represssor RNA and azide‐modified neomycin B (100 μm). All experiments were performed in triplicate. Median fluorescence is reported. AR=Anti‐repressor RNA; NeoB azide=azide‐modified neomycin B.

To assess whether the ligand‐mediated stabilization of the TMS switch solely depends on the interaction with the corresponding aptamer, we replaced the neomycin B aptamer of NeoB‐TMS^‐IBE^ with a kanamycin B aptamer^[30]^ (KanB‐TMS^‐IBE^). Azide‐modified neomycin B has a very low binding affinity for the kanamycin B aptamer (Figure S11). Indeed, due to the lower affinity of the kanamycin B aptamer for azide‐modified neomycin B, KanB‐TMS^‐IBE^ is unable to control gene expression with azide‐modified neomycin B (Figure S12). Hence, the ligand‐mediated structural changes in the TMS switch depend on the affinity of the ligand for the aptamer component of the switch.

We next asked whether the TMS switches can be deployed to target the bacterial genome. A d‐TMS^IBE^ switch was designed to target the T7 RNA polymerase gene present in the genome of the *E. coli* BL21(DE3) cells (Figure 5 a), binding from −30 to −1 bases upstream of the start codon in the corresponding mRNA. The d‐TMS^IBE^ switch is under control of the strong constitutive lpp promoter. To induce T7 polymerase expression, we added 0.1 mm isopropyl β‐d‐1‐thiogalactopyranoside (IPTG). As a marker, GFP expression from a plasmid controlled by a T7 promoter should be reduced when the d‐TMS^IBE^ switch represses T7 RNA polymerase translation. Indeed, we observed that the presence of the d‐TMS^IBE^ switch reduced GFP fluorescence (Figure 5 b). Importantly, binding cognate antirepressor RNA to the d‐TMS^IBE^ switch showed full GFP fluorescence, thus showing that genomic expression can also be switched (Figure S13). GFP expression is only marginally reduced further when a second d‐TMS^IBE^ switch that targets the +11 to +30 bases upstream of the start codon of the T7 polymerase mRNA is employed simultaneously with the first d‐TMS^IBE^, thus indicating the excellent performance of the switches.

**Figure 5:**
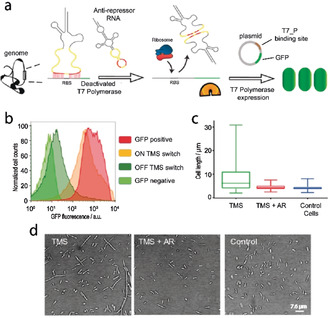
a) Switching genomic gene expression. The d‐TMS^IBE^ switch prevents expression of T7 RNA polymerase, by binding the −30 to −1 bases of the T7 polymerase gene. GFP expression is recovered with the cognate anti repressor RNA. b) Corresponding flow cytometry data showing repression of expression with d‐TMS^IBE^ (OFF TMS switch), activation with the anti‐repressor RNA (ON TMS switch), and controls as in Figure 2, but under a T7 promoter. c) Box plot showing that inhibiting a native *E. coli* gene ftsZ with two d‐TMS^IBE^ switches leads to filamentous growth compared to cells without any d‐TMS^IBE^ switch. Coexpression of the cognate anti‐repressor RNAs leads to restoration of cell sizes similar to d‐TMS^IBE^‐free cells. *n*=500 cells. d) Corresponding confocal microscopy brightfield images. Scale bar=7.6 μm. All experiments were performed in triplicate.

A key advantage of a trans‐encoded switch is that it allows genes in the genome to be targeted without sequence alteration of the genome. To demonstrate inhibition of native gene expression, we targeted the d‐TMS^IBE^ switch versus the ftsZ gene in the genome, which encodes for the FtsZ protein (Figure 5 d). FtsZ is an essential protein for cell division that forms a contractile ring structure (Z ring) at the future cell‐division site.^[31]^ One of the functions of the FtsZ ring is to recruit other cell division proteins to the septum to produce a new cell wall between the dividing cells.^[32]^ Inhibiting the FtsZ production would hamper the cell‐division process, leading to filamentous growth of *E. coli* cells.[^33^, ^34^] Unlike the T7 polymerase gene in the bacterial genome, the ftsZ gene is controlled by a constitutive promoter. Therefore, we decided to express two d‐TMS^IBE^ switches simultaneously to target the ftsZ gene in order to achieve tight control of FtsZ expression. One d‐TMS^IBE^ switch binds to the −19 to +11 nucleotides region and the other binds to the +32 to +61 nucleotides region from the start codon of the ftsZ gene. Transcription of both d‐TMS^IBE^ switches led to filamentous cells and a larger spread of cell lengths in comparison with cells without the switches (Figure 5 c; Figure S14). Both switches accept inputs from their respective cognate antirepressor RNAs, also expressed simultaneously, giving cells with the same average length and distribution as untreated cells. Hence, we can repress translation of mRNAs transcribed from both plasmid and genome without alteration of the target mRNA sequence, and subsequently turn on gene expression with input signals.

In summary, we present powerful new RNA‐based switches that provide unparalleled versatility in controlling bacterial gene expression. Unlike other cis‐acting genetic switches, the TMS switches do not pose sequence constrains on the target mRNA. By designing RNA switches that accept versatile inputs (RNA, small molecules, and proteins), we have been able to compensate for the low chemical diversity of input signals of the current RNA switches compared to their protein counterparts.^[35]^ A remarkable feature of the TMS switches is that the simple replacement of modules enables sensing of new input signals, and even addition of a second sensing module does not compromise performance of the RNA device as realized by the logic‐gate design. The switches that we developed here display a strong dynamic range, likely due to the stability of the TMS structure tuned by the unique design of the repressor domain. With their unique combination of signal processing of desired inputs and targeting any desired mRNA, the TMS switches will play an essential role in novel genetic circuitry to allow advanced information processing in synthetic biology.

## Conflict of interest

The authors declare no conflict of interest.

## Supporting information

As a service to our authors and readers, this journal provides supporting information supplied by the authors. Such materials are peer reviewed and may be re‐organized for online delivery, but are not copy‐edited or typeset. Technical support issues arising from supporting information (other than missing files) should be addressed to the authors.

Supplementary
