# Supplementary material for: Modular and Versatile Trans‐Encoded Genetic Switches
Source: Angew Chem Int Ed Engl. 2020 Jul 27;59(46):20328–32. doi: 10.1002/anie.202001372 (PMC7689881; doi:10.1002/anie.202001372)
Supplement: Supplementary file 1 — Supplementary [file ANIE-59-20328-s001.pdf]

Angewandte Chemie
Eine Zeitschrift der Gesellschaft Deutscher Chemiker

Supporting Information

Modular and Versatile Trans-Encoded Genetic Switches

*Avishek Paul, Eliza M. Warszawik, Mark Loznik, Arnold J. Boersma, and Andreas Herrmann**

anie_202001372_sm_miscellaneous_information.pdf

Supplementary methods

Supplementary Text

Supplementary Figure 1. The secondary structure of the TMS switch containing the repressor domain at the anti-codon arm.

Supplementary Figure 2. The plasmid maps of the TMS switch and GFP.

Supplementary Figure 3. Repression of the GFP fluorescence by the candidate TMS switches.

Supplementary Figure 4. Determination of the minimum length of the stem in the repressor domain, the stem that attaches the repressor domain with the tRNA structure and the variable loop required to maintain the functionality of the TMS switch.

Supplementary Figure 5. Stability of the TMS switch in RNase environment.

Supplementary Figure 6. The plasmid map of the Antirepressor.

Supplementary Figure 7. Secondary structures of the TMS switch and the NeomycinB aptamer.

Supplementary Figure 8. Isothermal titration calorimetry (ITC) test to measure the binding affinity between the NeomycinB aptamer and NeomycinB-azide.

Supplementary Figure 9. MIC test with NeomycinB-azide to determine its working concentration.

Supplementary Figure 10. MIC test with pristine NeomycinB.

Supplementary Figure 11. Isothermal titration calorimetry (ITC) test to measure the binding affinity between the KanamycinB aptamer and NeomycinB-azide.

Supplementary Figure 12. Study of TMS switch functionality in presence of NeomycinB and KanamycinB aptamers.

Supplementary Figure 13. Control expression of the T7 RNA polymerase gene by the TMS switch.

Supplementary Figure 14. Histograms of controlling FtsZ translation by the TMS switch.

Supplementary Sequences 1. Sequences of the different switches, origins of replication and the target GFP.

Supplementary Sequences 2. Sequences of eight different TMS switches with different length of A and B sub-domains against the target GFP sequence 5’TTAAATTGCTAAGGAGATGAAATC3’.

Supplementary Sequences 3. Five different sequences used to determine the minimum length of the stems and the variable loop of the TMS switch.

Supplementary Sequences 4. Sequence of the construct to express pristine repressor RNA.

Supplementary Sequences 5. Sequences used in the orthogonality test.

Supplementary Sequences 6. RNA sequences to target FtsZ and T7 polymerase genes.

Supplementary Reference

Methods

Plasmid construction. All the DNA fragments were purchased from Integrated DNA Technologies and the primers were acquired from Sigma-Aldrich. The fragments were inserted into the vector backbone through conventional cloning (Supplementary Ref. 1). The pBluescript vector backbone contained the DNA sequences of the TMS and anti-repressor switches. The reporter plasmid was based on the pZE21 vector backbone. The pBluescript vector encoded the pUC origin of replication (Supplementary Ref. 2) and the pZE21 vector contained p15A origin of replication (Supplementary Ref. 3). The pBluescript and the pZE21 plasmids contained ampicillin and chloramphenicol resistance genes, respectively. All constructs were transformed in *Escherichia coli* (*E. coli*) DH5α cells and were sequenced.

Growth of the *E. coli* cells and transcription of the TMS switches. To verify the function of the TMS switches, we used *E. coli* BL21(DE3) cells. Two separate tubes of the BL21(DE3) cells were used to execute the transformation process. In the first tube of BL21(DE3) cells, the TMS switch plasmid and the reporter plasmid were co-transformed. The anti-repressor RNA plasmid, which contains both the gene for the anti-repressor and TMS switch, was co-transformed with the reporter plasmid in the second tube of the cells. The transformation was carried out by electroporation (using MicroPulser Electroporator from BIO-RAD with 2.5 kV for 5 milliseconds). We spread the transformed cells on antibiotic plates, single colonies were picked and grown in LB media shaking at 200 rpm overnight at 37°C in the presence of antibiotics (ampicillin 50 µg/ml and chloramphenicol 25 µg/ml). The starter culture was then diluted by 200 times in LB medium containing antibiotics and four separate cultures were prepared – the first culture was to express the reporter gene only, the second culture was to express the switch and the reporter gene, the third culture was to express the switch, the reporter and the anti-repressor RNA and the fourth culture was used as a negative control. All the samples were grown at 37°C with 200 rpm to OD_{600} 0.4 – 0.6, after which 0.1% arabinose (w/v) was added into the first culture to induce the expression of the reporter gene only. Into the

second culture, 1mM IPTG and 0.1 % arabinose (w/v) were added to induce the switch and the reporter. Into the third culture, 1mM IPTG and 0.1 % arabinose (w/v) were added to induce the switch, the anti-repressor and the reporter. In the fourth culture, no inducer was added. After inducing each sample at the log phase, we incubated for six hours and then the output signal from each sample was measured by flow cytometry. To control gene expression by the TMS switch with protein as an input, we used mcherry as an output signal by replacing the GFP in the reporter plasmid with mcherry protein. The mcherry protein was placed under the control of an arabinose promoter. Here the GFP was used as an input signal to control the expression of the mcherry by the TMS switch. The GFP was cloned in the same reporter plasmid under a tet promoter. To study the gene expression with the TMS switch and protein as input, all the experimental procedure was same as mentioned above except the third culture was further divided into five separate sub-cultures. At the log phase 1 mM IPTG, 0.1% (w/v) arabinose were added into each five sub-cultures. Along with IPTG and arabinose, anhydrotetracycline was also added into those cultures with different concentrations (0.0125-0.2 µM). The flow cytometry measurement was taken after six hours of incubation.

Switching genomic FtsZ expression in *E. coli.* *E. coli* BL21(DE3) competent cells were either transformed with the TMS_FtsZ plasmid, or with both the TMS_FtsZ and TMS-AR_FtsZ plasmids: The TMS_FtsZ encoded the TMS switch that targets mRNA from the FtsZ gene, and the TMS-AR-FtsZ plasmid produced the corresponding anti-repressor switch. Expression of the switches was achieved under constitutive lpp promoters. Single colonies were grown in LB media shaking at 200 rpm at 37°C in the presence of antibiotic (ampicillin 50 µg/ml). The bacteria were analysed by fluorescence confocal microscopy upon reaching log phase (Leica TCS SP8, 63×water objective). Five µl cells were added on a glass slide and a cover slide was put on top. The images of the cells were analysed by ImageJ and the Feret diameter of each cell was taken as cell length. As control, BL21(DE3) cells without plasmid were grown, measured and analyzed in the same manner.

Flow cytometry measurements. Flow cytometry measurements were performed on a *BD FACS Canto* flow cytometer. The *BD FACS Canto* was calibrated with CST beads from BD Biosciences (Cat No: 655051). Samples were washed with 1X PBS and were diluted by 50-fold into 1X PBS buffer prior measurement. For GFP fluorescence measurement, 488 nm excitation filter (optical power 20mW) and 515-545 nm emission filter were used. For mcherry fluorescence measurement, 561 nm excitation filter (optical power 40mW) and 615-620 nm emission filter were used. For each sample, 50.000 events were recorded. All the samples were measured with low sample flow rate (approximately 12 µl/min). Cells were gated based on the positive fluorescence level. The flow cytometry analysis was performed using FlowJo software (version 10).

ITC. ITC experiments were executed using the ultrasensitive ITC 200 calorimeter (MicroCal) at 25°C. First, we degassed all solutions for 15 min using a vacuum pump in order to prevent the formation of bubbles in the sample cell during the experiment. We filled the reference cell with degassed distilled water and rinsed the sample cell with the buffer two times. We filled the sample cell with 7 µM aptamer solution (prepared in phosphate buffer solution with pH 6.8) and calorimeter syringe with 70 µM target ligand solution (prepared in phosphate buffer solution with pH 6.8). A purge-refill cycle was performed during filling up the syringe to avert the formation of air bubbles inside the syringe. To determine the binding constant, the ligand solution was added to the cell containing aptamer solution in a stepwise manner. The instruction of the instrument was set up in such a way that 20 injections of 2 µl volume from the syringe were added into the aptamer solution with intervals of 60 or 120 seconds between each injection. Control experiments were done by titrating the phosphate buffer without ligand into the aptamer solution. Data were analysed by using the nonlinear curve-fitting functions for one binding site provided by the ORIGIN software of MicoCal.

MIC. *E. coli* BL21(DE3) cells were grown in LB medium at 37 °C with 200 rpm until an OD_{600} of 0.6, after which they were diluted to OD_{600} 0.1 with fresh LB medium. NeomycinB-azide was serial diluted in

200 µL LB medium from 25.6 mM to 50 µM in 1 mL Deepwell 96-well plates (Eppendorf, Germany). 200 µL of the diluted *E.coli* culture was added to each well, diluting the NeomycinB-azide further from 12.8 mM to 25 µM. After 18 h incubation at 37 °C with 200 rpm, 200 µL of the culture was transferred to clear 96-well microtiter plates (Brand, Germany), and the turbidity was measured at 600 nm using a SpectraMax M3 platereader (Molecular Devices, USA). To conduct the MIC test for pristine NeomycinB, same procedure was adopted but the range of the serial dilution for NeomycinB was from 100 µM to 0.78 µM.

Secondary structure prediction of RNA. All secondary structure predictions of RNA were performed using the mfold Web Server (Supplementary Ref. 4).

Statistical analyses. All statistical analyses were performed using GraphPad Prism version 7.04 for Windows, GraphPad Software, La Jolla California USA, www.graphpad.com.

Data and plasmids availability. All the plasmids that have been used in this study and the raw data of flow cytometry and ITC will be available from the authors upon request.

Supplementary Text

Designing the TMS switch with region A = 10 nts and region B = 8 nts.

To design the repressor domain of the TMS switch, we first determined the secondary structure of the target region of the mRNA molecule. A region of 47 nts length of the target mRNA molecule has been chosen to make the design process straightforward. To design the A and B regions of the repressor domain, we took the reverse complementary sequences of the flanking regions of the Ribosome Binding Site (RBS) in the target mRNA. For region A, 10 nts reverse complementary region was chosen and for region B, 8 nts reverse

complementary region was selected. An ideal repressor domain should not impose any sequence constrains on the anti-repressor RNA and therefore we did not include the complementary sequence of the 6 nts RBS in the repressor domain. Instead, we incorporated random nucleotides in between the A and B regions of the repressor domain.

Next, we decided to include two 9 nts sequences of Initial Binding Elements (IBE) into the repressor domain, to provide initial binding sites between the repressor domain and the anti-repressor RNA. To attach the IBE to the repressor sequence, a stem of 8 nts length was incorporated into the structure. Finally, a stem of 4 nts length was chosen to attach the repressor domain to the respective tRNA structure.

After setting up these initial parameters, we used NUPACK software (Supplementary Ref. 5) to design the whole repressor domain and the stem that connects the repressor domain with the tRNA structure. In order to design these parts of the switch, the following algorithm was used:

```
#
# design material, temperature (C)
#
material = rna
temperature = 37.0
#
# target structures
#
structure hairpin1 = D4 (U9 D8 (U24) U9)
#
# sequence domains
#
domain a = N4
domain b = N9
domain c = N8
```

domain d = GAUUUCAUN6AGCAAUUUAA

domain e = N9

#

thread sequence domains onto target structures

#

hairpin1.seq = a b c d c* e a*

#

stop conditions for normalized ensemble defect

default: 1.0 (percent) for each target structure

In this algorithm, we specified the sequence of the A and B regions of the repressor domain and 6 random nts in “domain d”. For the design algorithm, 1.0 M Na^{+} and 0 M Mg^{+2} were selected as buffer condition. After executing the algorithm, the NUPACK software determined the sequences of the IBE domains, the stem that connects the repressor sequence to the IBE and the stem that connects the repressor domain to the tRNA structure.

The sequence of the IBE domains was the following:

5’ AAAAUAAGA 3’

5’ GAAGCCAGA 3’

The sequence of the stem that connects the IBE to the repressor sequence was the following:

5’ UCGAGUCG

3’ AGCUCAGC

The sequence of the stem that connects the repressor domain to the tRNA structure was the following:

5’ GCGG

3’ CGCC

We also determined the free energy of the structural complex formed by the binding of the repressor domain and the target mRNA by executing the following algorithm in the NUPACK software,

```
#
# design material, temperature (C)
#
material = rna
temperature = 37.0
#
# target structure
#
structure stickfigure = D10 (U6 D8 (+) U6)
#
# sequence domains
#
domain a = GAUUUCAUCUGCAAAGCAAUUUAA
domain b = N8AAGGAGN10
#
# strands (used for threading sequence information
# and for displaying results)
#
strand left = a
strand right = b
#
# thread strand sequence information onto target structures
#
```

stickfigure.seq = left right

#

specify stop conditions for normalized ensemble defect

default: 1.0 (percent) for each target structure

#

stickfigure.stop = 1.0

The free energy of the structural complex was calculated to be -22.18 kcal/mol. The software also allowed to obtain the average number of nucleotides (2.9 nts) in the complex that could be incorrectly paired at the equilibrium relative to the specified secondary structure (the number was evaluated over the Boltzman-weighted ensemble of secondary structures). The normalized ensemble defect was 4.4%.

We also used NUPACK software to design the different repressor domains used in the orthogonality test. To conduct the orthogonality test, we designed six different TMS switches with the same stem sequence that connects the repressor domains to the tRNA structure. The TMS switches differ in the sequences of IBE domains and the stem that connects the IBE to the repressor sequence. They also contain different sequences in the A and B regions of the repressor domain. After setting up these parameters we executed the following algorithm:

#

design material, temperature (C)

#

material = rna

temperature = 37.0

#

target structures

#

structure hairpin1 = D4 (U9 D8 (U24) U9)

#

sequence domains

#

domain a = GCGG

domain b = N9

domain c = N8

domain d = N24

domain e = N9

#

thread sequence domains onto target structures

#

hairpin1.seq = a b c d c* e a*

#

stop conditions for normalized ensemble defect

default: 1.0 (percent) for each target structure

#

prevent sequence patterns

#

prevent = AAAA, CCCC, GGGG, UUUU, KKKKKK, MMMMMM, RRRRRR, SSSSSS, WWWWWW, YYYYYY

After executing the algorithm, the NUPACK software provided different sequences of the TMS switches where each TMS switch contained a specific repressor domain sequence.

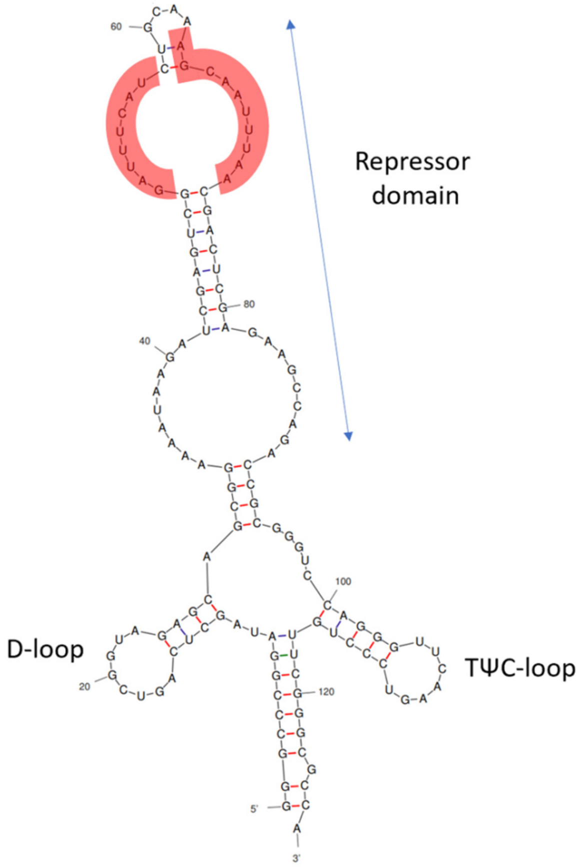

Figure S1. The secondary structure of the TMS switch containing the repressor domain at the anti-codon arm. The nucleotides which are modified during tRNA processing are denoted by red circles. Target sequence of the GFP mRNA is 5'TTAAATTGCTAAGGAGATGAAATC3'. The secondary structure of the TMS switch was determined by mfold web server. Red colour represents the binding domains of the TMS switch.

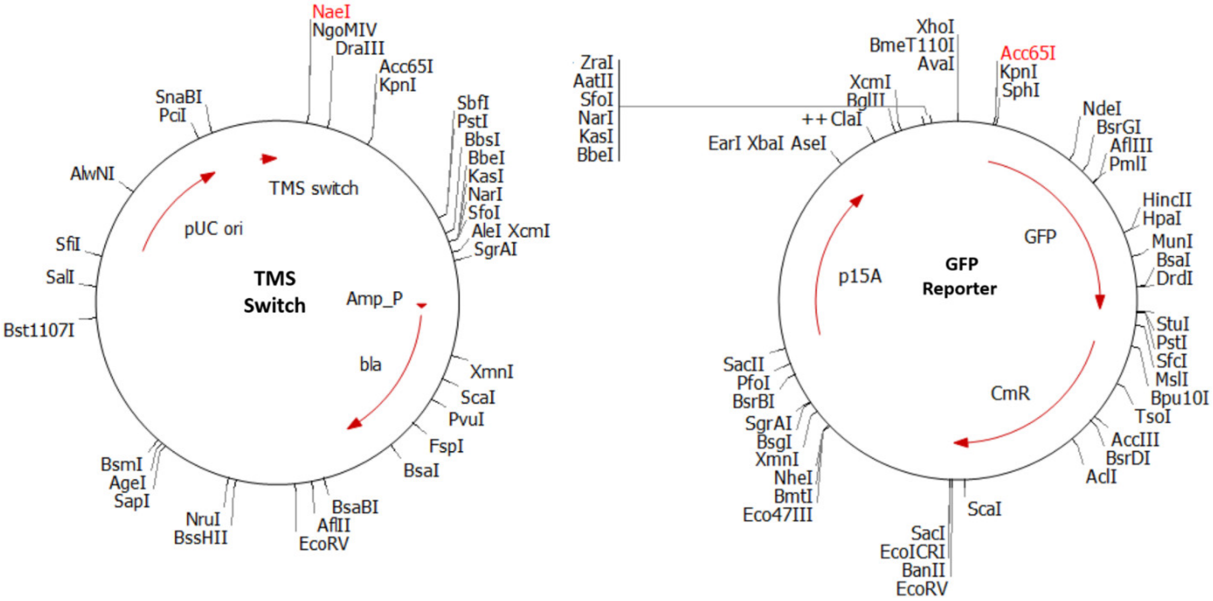

Figure S2. The plasmid maps of the TMS switch and GFP.

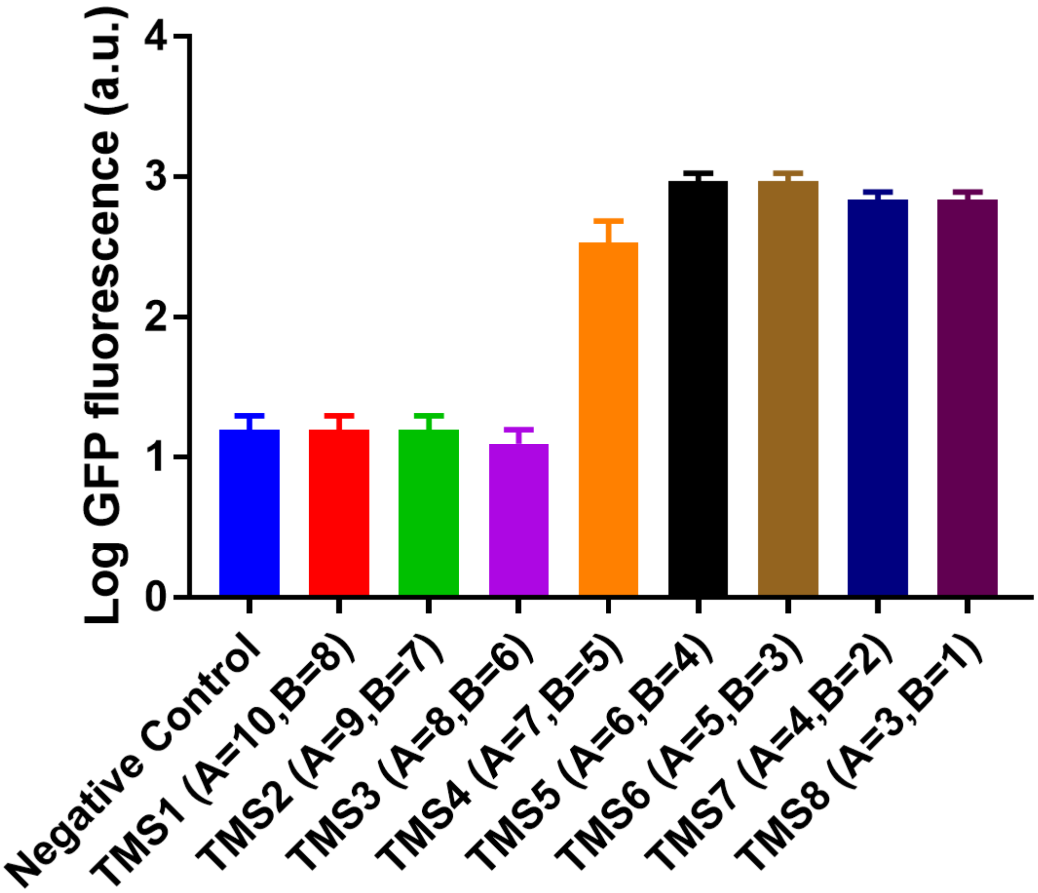

Figure S3. Repression of the GFP fluorescence by the candidate TMS switches. We designed eight different TMS switches, where each TMS switch contains a unique length of A and B regions (the two subdomains located in the repressor domain). We designed TMS1 switch in such a way that it contains region A of 10 nts and region B of 8 nts. We constructed the subsequent TMS switches by reducing the length of the A and B regions stepwise by 1 nt. We noticed that TMS switch number 1, 2 and 3 displayed GFP repression capability after 6 hours of incubation. The experiment was performed in triplicate (sequences of the eight different TMS switches are available in supplementary sequences 2). GFP fluorescence on Y axis represents the GFP fluorescence values in log scale.

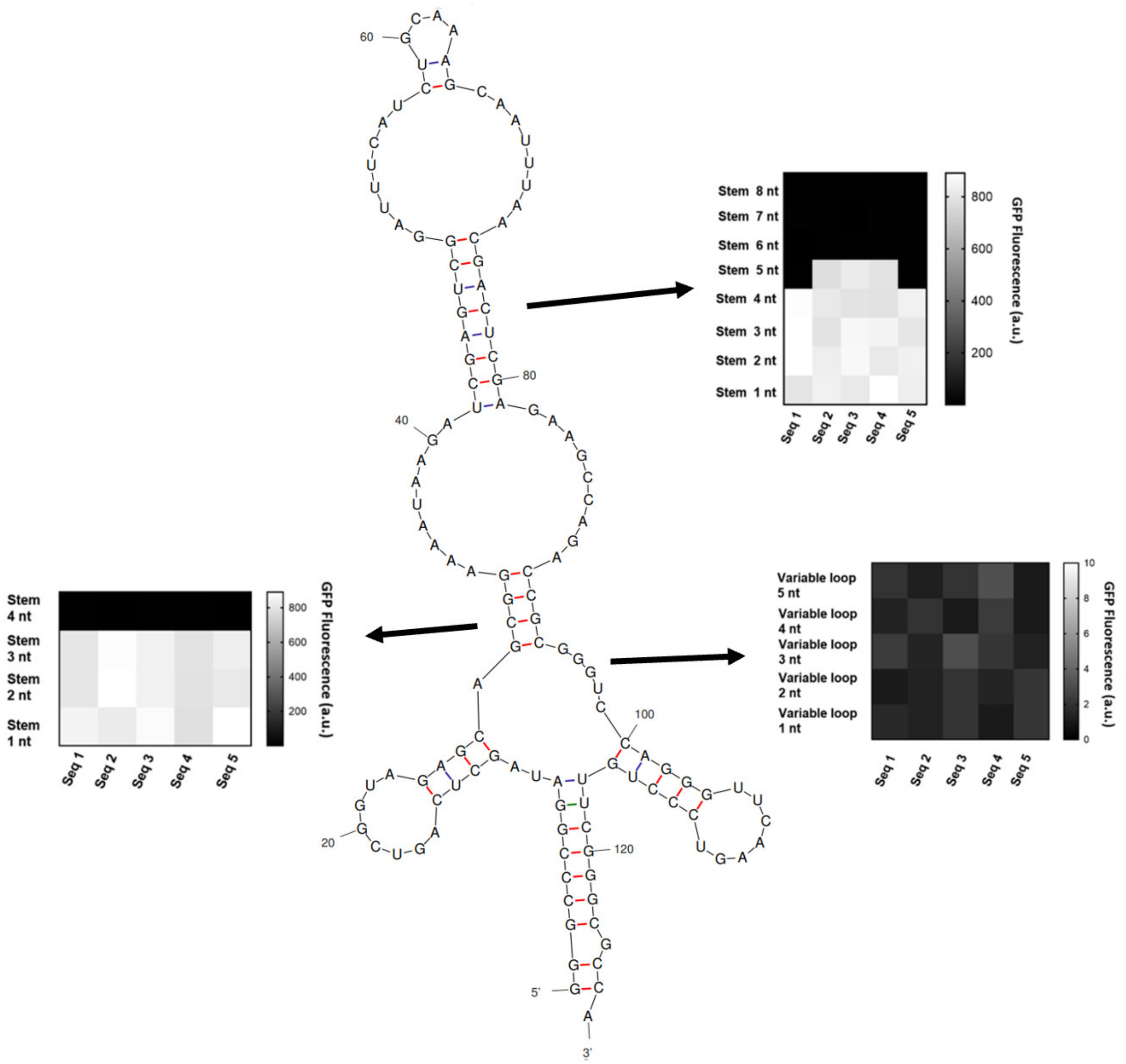

Figure S4. Determination of the minimum length of the stem in the repressor domain, the stem that attaches the repressor domain with the tRNA structure and the variable loop required to maintain the functionality of the TMS switch. We designed five different TMS switches with different length of the stem in the repressor domain (from 1 nt to 8 nts) and measured the median GFP for each candidate TMS switch against five corresponding reverse complementary target mRNA sequences. Similarly, we changed the length of the stem connecting the repressor with the tRNA structure in the five TMS switches and measured the median GFP intensity against the same five different target sequences that were used

previously to characterize the stem length of the repressor domain. In the same way, we changed the variable loop length of the five TMS switches and determined the median GFP intensity with the same five target sequences (the sequences of the five TMS switches and their target sequences are available in supplementary sequences 2).

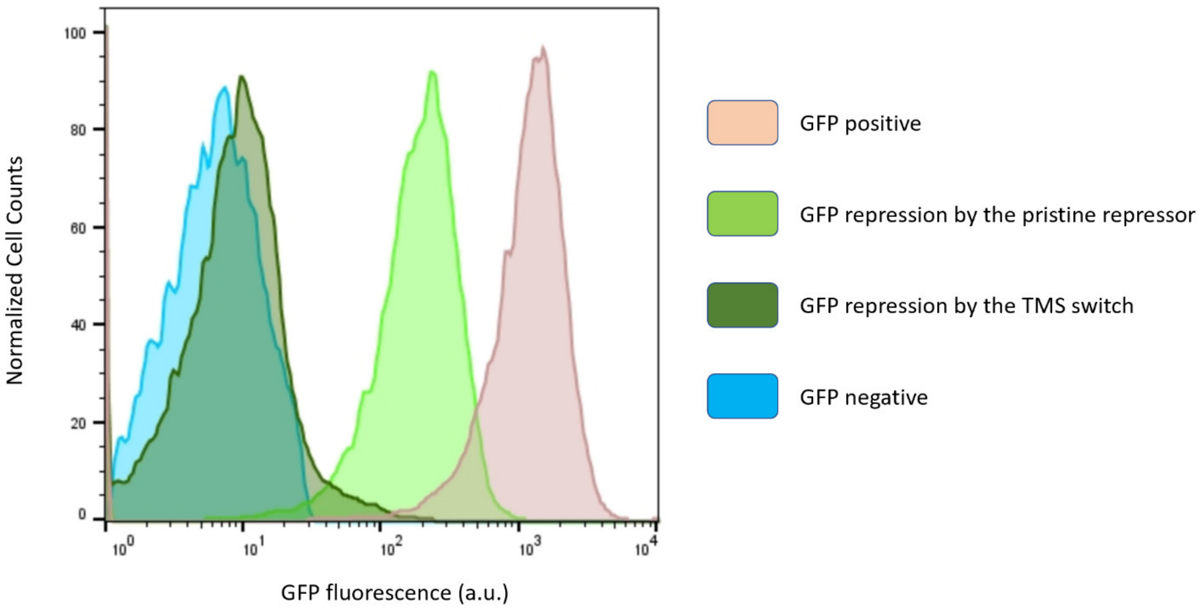

Figure S5. Stability of the TMS switch in RNase environment. GFP positive cells contain only the GFP reporter plasmid which was induced by arabinose and GFP negative cells contain GFP reporter plasmid but without any inducer. GFP repression by the pristine repressor refers to the repression of the GFP with only the repressor sequence without any tRNA scaffold. GFP repression by the TMS switch refers to the repression of the GFP with the repressor sequence incorporated into the tRNA scaffold.

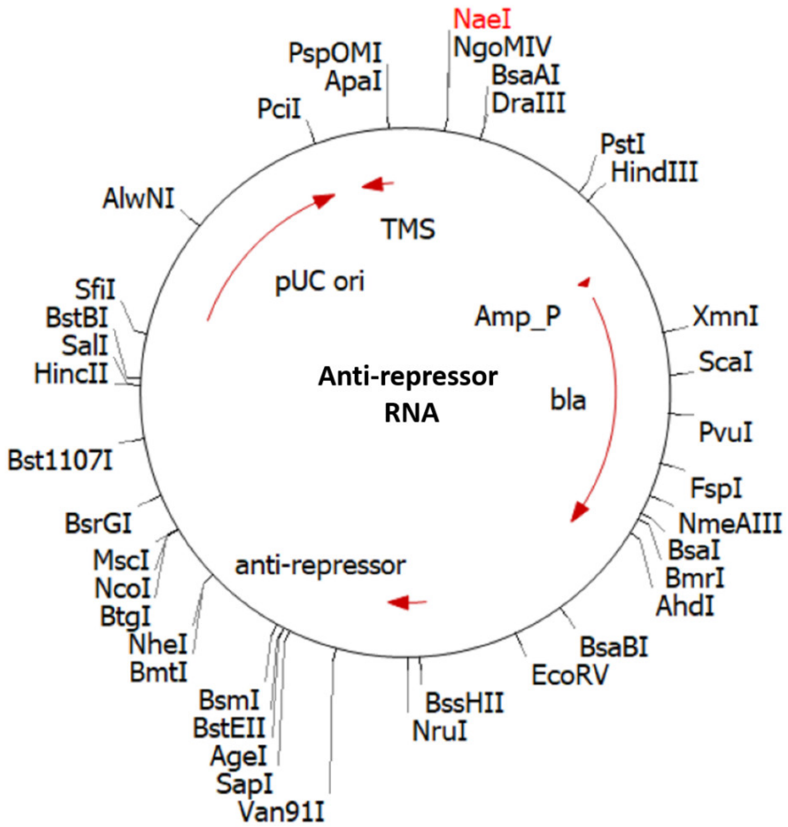

Figure S6. The plasmid map of the Antirepressor.

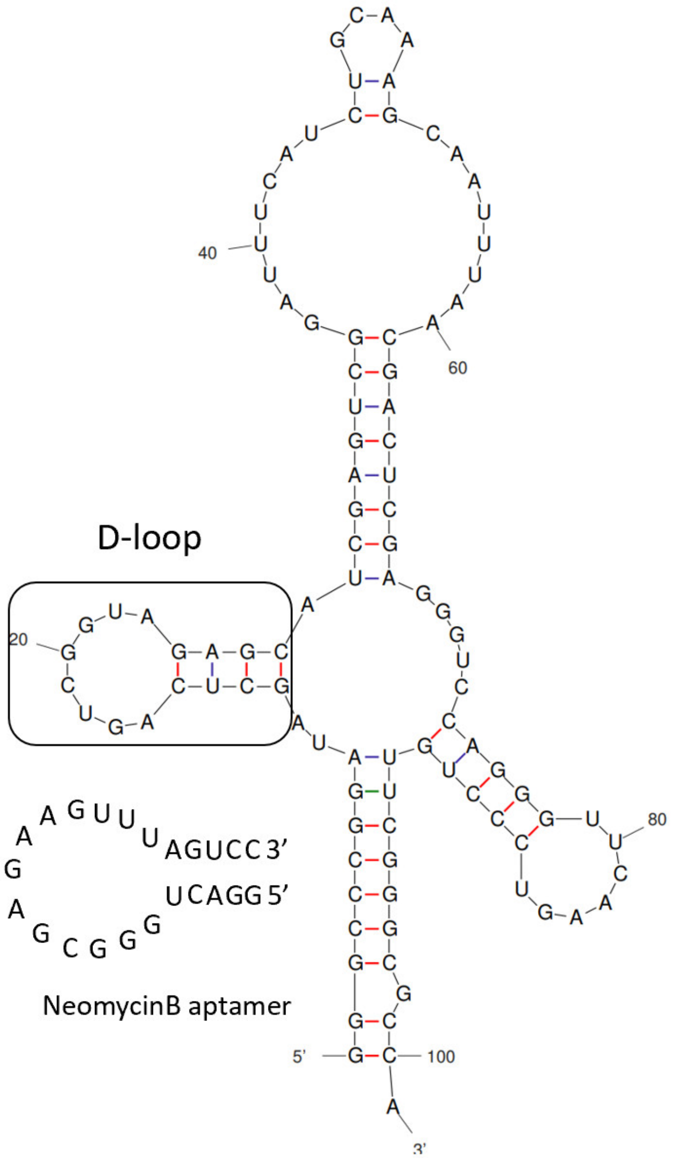

Figure S7. Secondary structures of the TMS switch and the NeomycinB aptamer. The aptamer was incorporated into the D-loop of the TMS structure without any sequence modification of the NeomycinB aptamer.

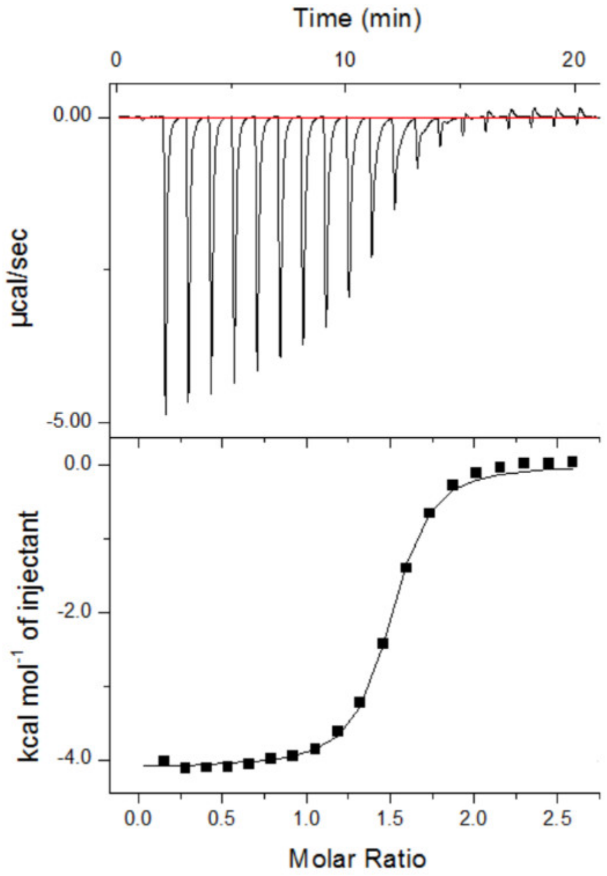

Figure S8. Isothermal titration calorimetry (ITC) test to measure the binding affinity between the NeomycinB aptamer and NeomycinB-azide. Analysis of the ITC binding curve showed a binding affinity of 28 μM. The sequence of the NeomycinB aptamer: 5'GGACTGGGCGAGAAGTTTAGTCC3'

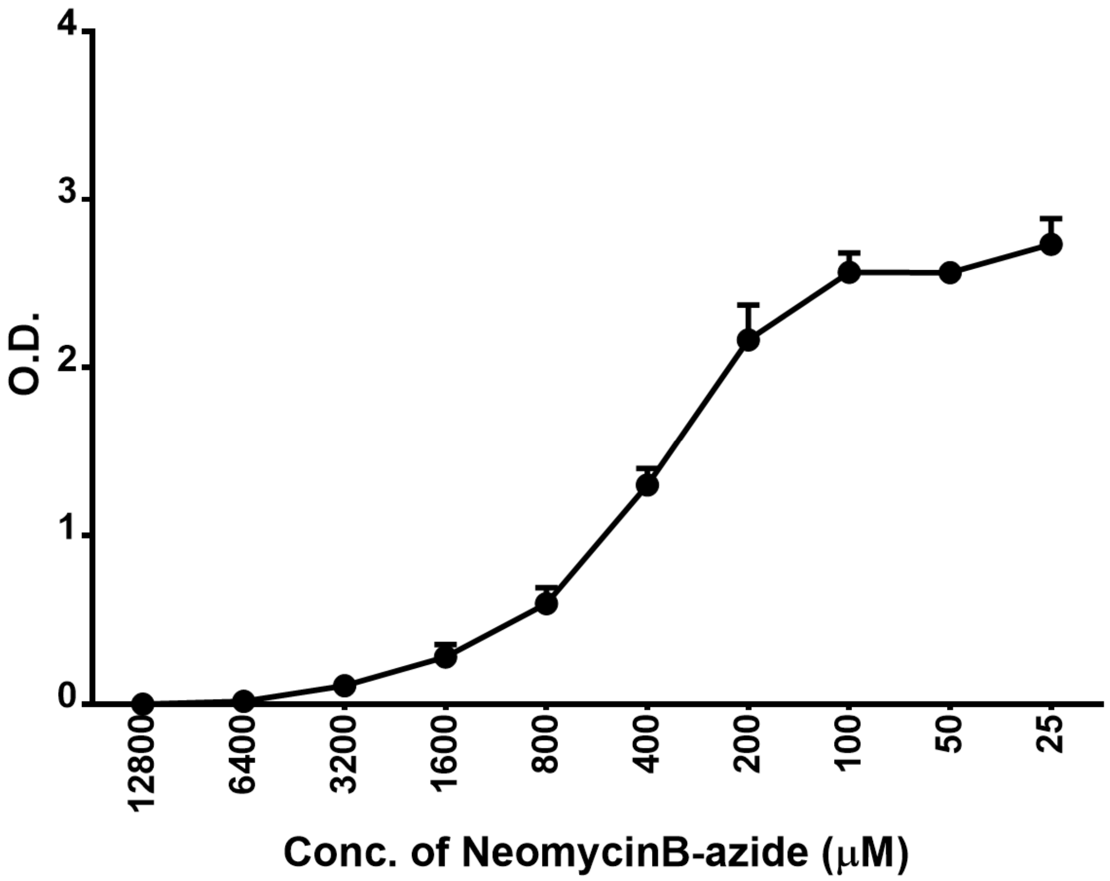

Figure S9. MIC test with NeomycinB-azide to determine its working concentration. We noticed from the MIC test that concentration higher than 100 μM of NeomycinB-azide disturbs bacterial growth. Therefore, we picked 100 μM NeomycinB-azide concentration to verify the TMS switch activity against the NeomycinB-azide.

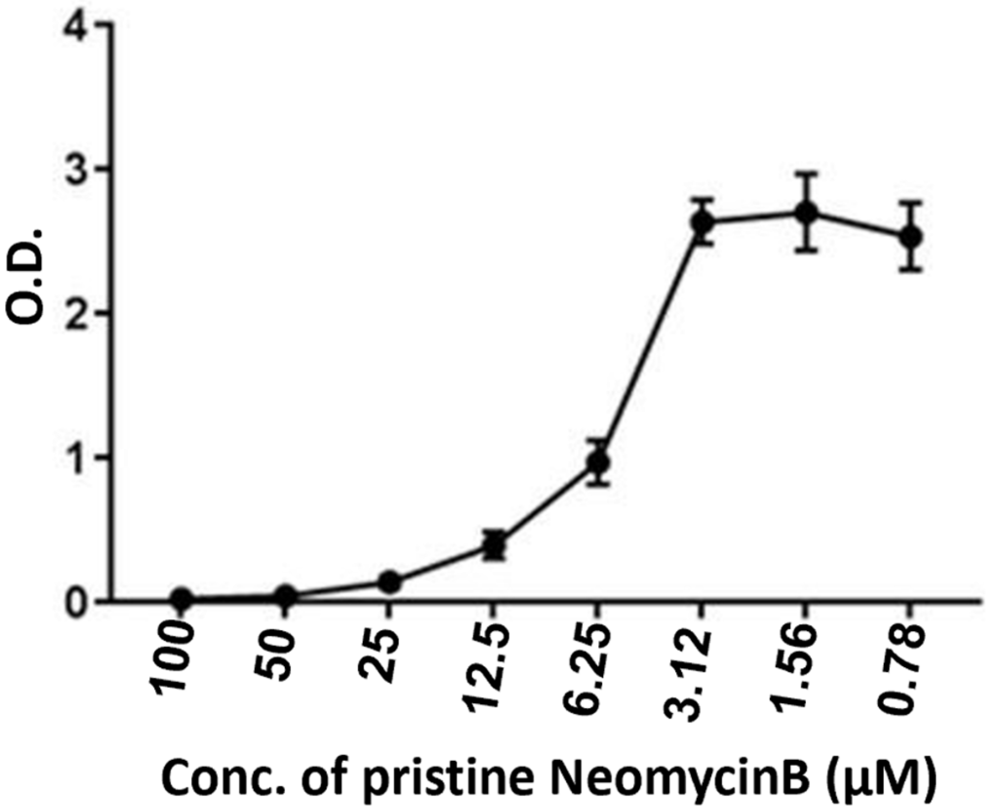

Figure S10. MIC test with pristine NeomycinB. We noticed from the MIC test that concentrations higher than 3.12 μM of NeomycinB can disturb bacterial growth.

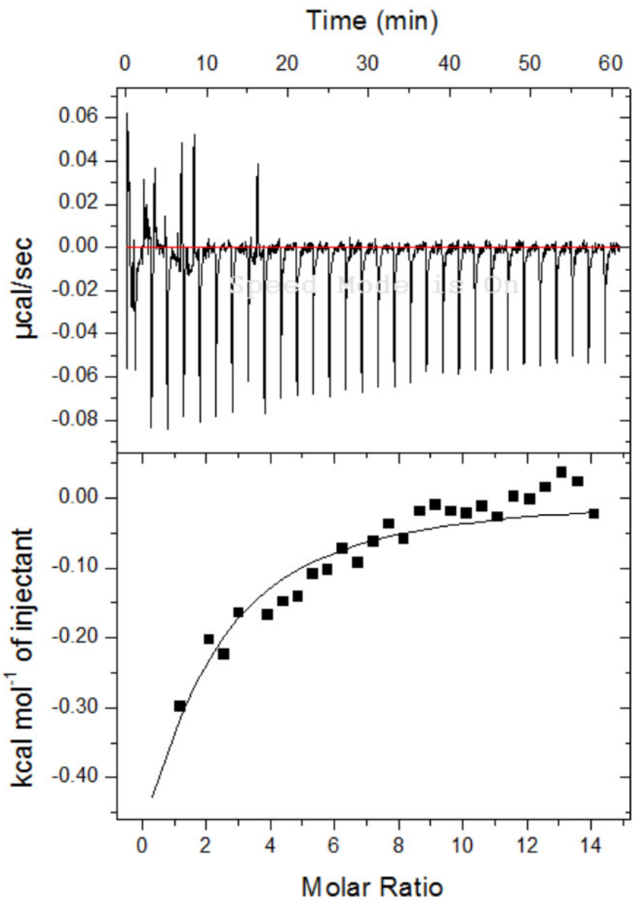

Figure S11. Isothermal titration calorimetry (ITC) test to measure the binding affinity between the KanamycinB aptamer and NeomycinB-azide. Analysis of the ITC binding curve showed a binding affinity of 2 mM. The sequence of the KanamycinB aptamer: 5'GGGAGCUCGGUACCGAAUUCUC3'

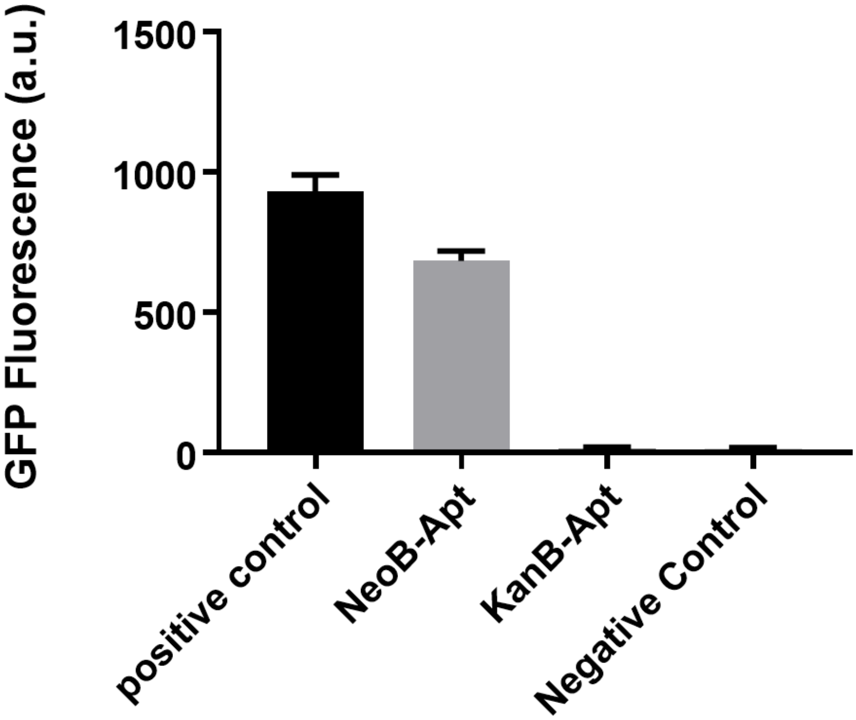

Figure S12. Study of TMS switch functionality in presence of NeomycinB and KanamycinB aptamers. Two separate TMS switch were prepared by replacing the D-loop of the tRNA with the respective aptamers. To check the functionality of each switches, NeomycinB-azide (100 μM) was used as target ligand.

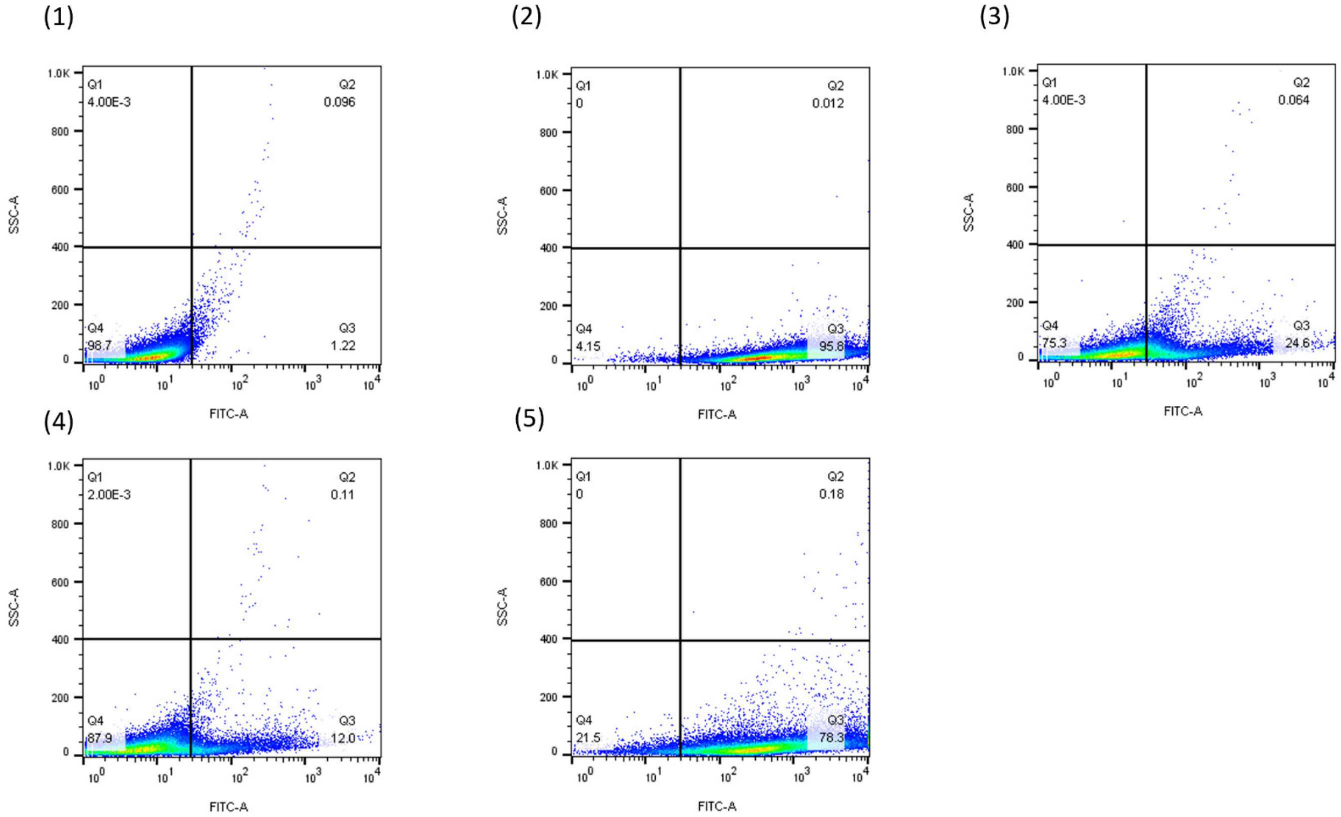

Figure S13. Control expression of the T7 RNA polymerase gene by the TMS switch. TMS switch was designed to target the mRNA of the T7 RNA polymerase gene and thus affects the expression of a GFP protein, which was under the control of a T7 promoter. (1) Negative control (without addition of IPTG); (2) Positive control (with addition of IPTG); (3) Single TMS switch targeting the mRNA of the T7 RNA polymerase gene and thereby reducing the GFP expression from the T7 promoter; (4) Dual TMS switch

targeting the mRNA of the T7 RNA polymerase gene; (5) Simulaneous expression of the TMS switch and its cognate anti-repressor RNA.

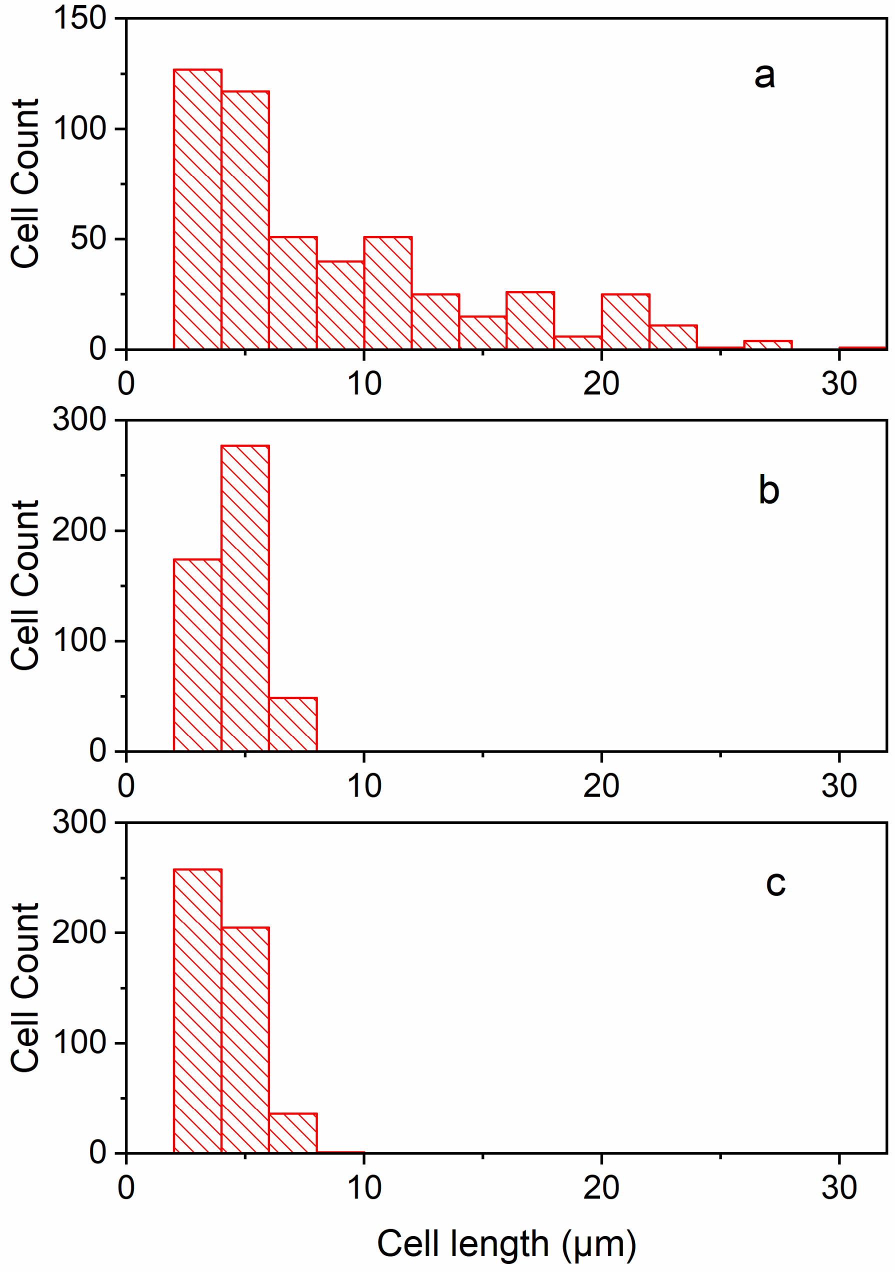

Figure S14. Histograms of controlling FtsZ translation by the TMS switch. (a) Cells expressing only the TMS switches; (b) Cells expressing both TMS switches and their cognate antirepressor RNAs; (c) Cells that neither express TMS switches nor antirepressor RNAs.

Supplementary Sequences 1. Sequences of the different switches, origins of replication and the target GFP

T7P = T7 Promoter

T7 TT = T7 transcription terminator

**araC-pBAD-binding region A-RBS-binding region B-GFP**	5'TTATGACAACTTGACGGCTACATCATTCACTTTTTCTTCACAACCGG<br>CACGGAACTCGCTCGGGCTGGCCCCGGTGCATTTTTTAAATACCCGC<br>GAGAAATAGAGTTGATCGTCAAAACCAACATTGCGACCGACGGTGG<br>CGATAGGCATCCGGGTGGTGCTCAAAAGCAGCTTCGCCTGGCTGATA<br>CGTTGGTCCTCGCGCCAGCTTAAGACGCTAATCCCTAACTGCTGGCG<br>GAAAAGATGTGACAGACGCGACGGCGACAAGCAAACATGCTGTGCG<br>ACGCTGGCGATATCAAAATTGCTGTCTGCCAGGTGATCGCTGATGTA<br>CTGACAAGCCTCGCGTACCCGATTATCCATCGGTGGATGGAGCGACT<br>CGTTAATCGCTTCCATGCGCCGCAGTAACAATTGCTCAAGCAGATTT<br>ATCGCCAGCAGCTCCGAATAGCGCCCTTCCCCTTGCCCGGCGTTAAT<br>GATTTGCCCAAACAGGTCGCTGAAATGCGGCTGGTGCGCTTCATCCG<br>GGCGAAAGAACCCCGTATTGGCAAATATTGACGGCCAGTTAAGCCA<br>TTCATGCCAGTAGGCGCGCGGACGAAAGTAAACCCACTGGTGATAC<br>CATTCGCGAGCCTCCGGATGACGACCGTAGTGATGAATCTCTCCTGG<br>CGGGAACAGCAAAATATCACCCGGTCGGCAAACAAATTCTCGTCCCT<br>GATTTTTCACCACCCCCTGACCGCGAATGGTGAGATTGAGAATATAA<br>CCTTTCATTCCCAGCGGTCGGTCGATAAAAAAATCGAGATAACCGTT<br>GGCCTCAATCGGCGTTAAACCCGCCACCAGATGGGCATTAAACGAGT

	ATCCCGGCAGCAGGGGATCATTTTGCGCTTCAGCCATACTTTTCATACTCCCGCCATTCAGAGAAGAAACCAATTGTCCATATTGCATCAGACATTGCCGTCACTGCGTCTTTTACTGGCTCTTCTCGCTAACCAAACCGGTAACCCCGCTTATTAAAAGCATTCTGTAACAAAGCGGGACCAAAGCCATGACAAAAACGCGTAACAAAAGTGTCTATAATCACGGCAGAAAAGTCCACATTGATTATTTGCACGGCGTCACACTTTGCTATGCCATAGCATTTTTATCCATAAGATTAGCGGATCCTACCTGACGCTTTTTATCGCAACTCTCTACTGTTTCTCCATAAGAACAATCCTCAACCAAGGAAAGGAGGTGAAAACACATGCGTAAAGGCGAAGAGCTGTTCACTGGTGTCGTCCCTATTCTGGTGGAACTGGATGGTGATGTCAACGGTCATAAGTTTTCCGTGCGTGGCGAGGGTGAAGGTGACGCAACTAATGGTAAACTGACGCTGAAGTTCATCTGTACTACTGGTAAACTGCCGGTACCTTGGCCGACTCTGGTAACGACGCTGACTTATGGTGTTCAGTGCTTTGCTCGTTATCCGGACCATATGAAGCAGCATGACTTCTTCAAGTCCGCCATGCCGGAAGGCTATGTGCAGGAACGCACGATTTCCTTTAAGGATGACGGCACGTACAAAACGCGTGCGGAAGTGAAATTTGAAGGCGATACCCTGGTAAACCGCATTGAGCTGAAAGGCATTGACTTTAAAGAAGACGGCAATATCCTGGGCCATAAGCTGGAATACAATTTTAACAGCCACAATGTTTACATCACCGCCGATAAACAAAAAAATGGCATTAAAGCGAATTTTAAAATTCGCCACACGTGGAGGATGGCAGCGTGCAGCTGGCTGATCACTACCAGCAAAACACTCCAATCGGTGATGGTCCTGTTCTGCTGCCAGACAATCACTATCTGAGCACGCAAAGCGTTCTGTCTAAAGATCCGAACGAGAAACGCGATCATATGGTTCTGCTGGAGTTCGTAACCGCAGCGGGCATCACGCATGGTATGGATGAACTGTACAAATGA3’

**T7P-TMS-binding region A-TMS-binding region B-T7 TT**	5'TAATACGACTCACTATAGGGCCCGGATAGCTCAGTCGGTAGAGCA<br>GCGGAAAATAAGATCGAGTCGGTTTTCACTTCCAATCCTTGGTTGCG<br>ACTCGAGAAGCCAGACCGCGGGTCCAGGGTTCAAGTCCCTGTTCGGG<br>CGCCACTAGCATAACCCCTTGGGGCCTCTAAACGGGTCTTGAGGGGT<br>TTTTTGGA3'
**T7P-TMS- Neo Apt- TMS-binding region A-TMS-binding region B -T7 TT**	5'TAATACGACTCACTATAGGGCCCGGATAGGACTGGGCGAGAAGTT<br>TAGTCCATCGAGTCGGTTTTCACCTGCAATCCTTGGTTGCGACTCGAG<br>GGTCCAGGGTTCAAGTCCCTGTTCGGGCGCCACTAGCATAACCCCTT<br>GGGGCCTCTAAACGGGTCTTGAGGGGTTTTTGGA3'
**T7P-TMS-GFP Apt-TMS-binding region A-TMS-binding region B -T7 TT**	5'TAATACGACTCACTATAGGGCCCGGATAGGACAGATCTGGGAGCA<br>CGATGGCGTGGCGAATTGGGTGGGGAAAGTCCTTAAAAGAGGGCCA<br>CCACAGAAGCAATGGGCTTCTGGACTCGGTAGATCTGTCCTCGAGTC<br>GGTTTTCACCTGCAATCCTTGGTTGCGACTCGAGGGTCCAGGGTTCA<br>AGTCCCTGTTCGGGCGCCACTAGCATAACCCCTTGGGGCCTCTAAAC<br>GGGTCTTGAGGGGTTTTTGGA3'
**T7P-Anti repressor RNA-T7 TT**	5'TAATACGACTCACTATAGGGCCCGGATAGCTCAGTCGGTAGAGCA<br>GCGGTCTGGCTTCTCGAGTCGCAACCAAGGATTGGAAGTGAAAACC<br>GACTCGATCTTATTTTCCGCGGGTCCAGGGTTCAAGTCCCTGTTCGGG<br>CGCCACTAGCATAACCCCTTGGGGCCTCTAAACGGGTCTTGAGGGGT<br>TTTTTGGA3'
**Ptet/pLtet-GFP-tetR-araC-pBAD-binding region A-RBS-binding region B-mcherry**	5'CTCGAGTCCCTATCAGTGATAGAGATTGACATCCCTATCAGTGATA<br>GAGATACTGAGCACATCAGCAGGACGCACTGACCGAATTCATTAAA<br>TTTAACTTTAAGAAGGAGATATACATATGCGTAAAGGCGAAGAGCT<br>GTTCACTGGTGTCGTCCCTATTCTGGTGGAACTGGATGGTGATGTCA

	ACGGTCATAAGTTTTCCGTGCGTGGCGAGGGTGAAGGTGACGCAACT<br>AATGGTAAACTGACGCTGAAGTTCATCTGTACTACTGGTAAACTGCC<br>GGTACCTTGGCCGACTCTGGTAACGACGCTGACTTATGGTGTTCAGT<br>GCTTTGCTCGTTATCCGGACCATATGAAGCAGCATGACTTCTTCAAG<br>TCCGCCATGCCGGAAGGCTATGTGCAGGAACGCACGATTTCCTTTAA<br>GGATGACGGCACGTACAAAACGCGTGCGGAAGTGAAATTTGAAGGC<br>GATACCCTGGTAAACCGCATTGAGCTGAAAGGCATTGACTTTAAAGA<br>AGACGGCAATATCCTGGGCCATAAGCTGGAATACAATTTTAACAGCC<br>ACAATGTTTACATCACCGCCGATAAACAAAAAAATGGCATTAAAGC<br>GAATTTTAAAATTCGCCACACGTGGAGGATGGCAGCGTGCAGCTGGC<br>TGATCACTACCAGCAAAACACTCCAATCGGTGATGGTCCTGTTCTGC<br>TGCCAGACAATCACTATCTGAGCACGCAAAGCGTTCTGTCTAAAGAT<br>CCGAACGAGAAACGCGATCATATGGTTCTGCTGGAGTTCGTAACCGC<br>AGCGGGCATCACGCATGGTATGGATGAACTGTACAAATGATACAAA<br>GATGCATGCCAGTTCTAGCATAACCCTAATGAGTGAGCTAACTTACA<br>TTAATTGCGTTGCGCCTTAATTAACGGCACTCCTCAGCAAATATAAT<br>GACCCTCTTGATAACCCAAGAGGGCATTTTTTAATGCCCATGGCGTT<br>TACCACAGCTAACACCACGTCGTCCCTATCTGCTGCCCTAGGTCTAT<br>GAGTGGTTGCTGGATAACTTTACGGGCATGCATAAGGCTCGTAGGCT<br>ATATTCAGGGAGACCACAACGGTTTCCCTCTACAAATAATTTTGTTT<br>AACTTTGAAATAAGGAGGTAATACAAATGTCTCGTTTAGATAAAAGT<br>AAAGTGATTAACAGCGCATTAGAGCTGCTTAATGAGGTCGGAATCG<br>AAGGTTTAACAACCCGTAAACTCGCCCAGAAGCTAGGTGTAGAGCA<br>GCCTACATTGTATTGGCATGTAAAAAATAAGCGGGCTTTGCTCGACG<br>CCTTAGCCATTGAGATGTTAGATAGGCACCATACTCACTTTTGCCCTT

	TAGAAGGGGAAAGCTGGCAAGATTTTTTACGTAATAACGCTAAAAG
	TTTTAGATGTGCTTTACTAAGTCATCGCGATGGAGCAAAAGTACATT
	TAGGTACACGGCCTACAGAAAAACAGTATGAAACTCTCGAAAATCA
	ATTAGCCTTTTTATGCCAACAAGGTTTTTCACTAGAGAATGCATTATA
	TGCACTCAGCGCTGTGGGGCATTTTACTTTAGGTTGCGTATTGGAAG
	ATCAAGAGCATCAAGTCGCTAAAGAAGAAAGGGAAACACCTACTAC
	TGATAGTATGCCGCCATTATTACGACAAGCTATCGAATTATTTGATC
	ACCAAGGTGCAGAGCCAGCCTTCTTATTCGGCCTTGAATTGATCATA
	TGCGGATTAGAAAAACAACTTAAATGTGAAAGTGGGTCTTAATTATG
	ACAACTTGACGGCTACATCATTCACTTTTTCTTCACAACCGGCACGG
	AACTCGCTCGGGCTGGCCCCGGTGCATTTTTTAAATACCCGCGAGAA
	ATAGAGTTGATCGTCAAAACCAACATTGCGACCGACGGTGGCGATA
	GGCATCCGGGTGGTGCTCAAAAGCAGCTTCGCCTGGCTGATACGTTG
	GTCCTCGCGCCAGCTTAAGACGCTAATCCCTAACTGCTGGCGGAAAA
	GATGTGACAGACGCGACGGCGACAAGCAAACATGCTGTGCGACGCT
	GGCGATATCAAAATTGCTGTCTGCCAGGTGATCGCTGATGTACTGAC
	AAGCCTCGCGTACCCGATTATCCATCGGTGGATGGAGCGACTCGTTA
	ATCGCTTCCATGCGCCGCAGTAACAATTGCTCAAGCAGATTTATCGC
	CAGCAGCTCCGAATAGCGCCCTTCCCCTTGCCCGGCGTTAATGATTT
	GCCCAAACAGGTCGCTGAAATGCGGCTGGTGCGCTTCATCCGGGCGA
	AAGAACCCCGTATTGGCAAATATTGACGGCCAGTTAAGCCATTCATG
	CCAGTAGGCGCGCGGACGAAAGTAAACCCACTGGTGATACCATTCG
	CGAGCCTCCGGATGACGACCGTAGTGATGAATCTCTCCTGGCGGGAA
	CAGCAAAATATCACCCGGTCGGCAAACAAATTCTCGTCCCTGATTTT
	TCACCACCCCCTGACCGCGAATGGTGAGATTGAGAATATAACCTTTC

	ATTCCCAGCGGTCGGTCGATAAAAAAATCGAGATAACCGTTGGCCTC<br>AATCGGCGTTAAACCCGCCACCAGATGGGCATTAAACGAGTATCCCG<br>GCAGCAGGGGATCATTTTGCGCTTCAGCCATACTTTTCATACTCCCGC<br>CATTCAGAGAAGAAACCAATTGTCCATATTGCATCAGACATTGCCGT<br>CACTGCGTCTTTTACTGGCTCTTCTCGCTAACCAAACCGGTAACCCCG<br>CTTATTAAAAGCATTCTGTAACAAAGCGGGACCAAAGCCATGACAA<br>AAACGCGTAACAAAAGTGTCTATAATCACGGCAGAAAAGTCCACAT<br>TGATTATTTGCACGGCGTCACACTTTGCTATGCCATAGCATTTTTATC<br>CATAAGATTAGCGGATCCTACCTGACGCTTTTTATCGCAACTCTCTAC<br>TGTTTCTCCATAAGAACAATCCTCAACCAAGGAAAGGAGGTGAAAA<br>CACATGGTGAGCAAGGGCGAGGAGGATAACATGGCCATCATCAAGG<br>AGTTCATGCGCTTCAAGGTGCACATGGAGGGCTCCGTGAACGGCCAC<br>GAGTTCGAGATCGAGGGCGAGGGCGAGGGCCGCCCCTACGAGGGCA<br>CCCAGACCGCCAAGCTGAAGGTGACCAAGGGTGGCCCCCTGCCC<br>TTCGCCTGGGACATCCTGTCCCCTCAGTTCATGTACGGCTCCAAGGC<br>CTACGTGAAGCACCCCGCCGACATCCCCGACTACTTGAAGCTGTCCT<br>TCCCCGAGGGCTTCAAGTGGGAGCGCGTGATGAACTTCGAGGACGG<br>CGGCGTGGTGACCGTGACCCAGGACTCCTCCCTGCAGGACGGCGAGT<br>TCATCTACAAGGTGAAGCTGCGCGGCACCAACTTCCCCTCCGACGGC<br>CCCGTAATGCAGAAGAAGACCATGGGCTGGGAGGCCTCCTCCGAGC<br>GGATGTACCCCGAGGACGGCGCCCTGAAGGGCGAGATCAAGCAGAG<br>GCTGAAGCTGAAGGACGGCGGCCACTACGACGCTGAGGTCAAGACC<br>ACCTACAAGGCCAAGAAGCCCGTGCAGCTGCCCGGCGCCTACAACG<br>TCAACATCAAGTTGGACATCACCTCCCACAACGAGGACTACACCATC

	GTGGAACAGTACGAACGCGCCGAGGGCCGCCACTCCACCGGCGGCA TGGACGAGCTGTACAAGTAG3’
**pUC**	CCGTAGAAAAGATCAAAGGATCTTCTTGAGATCCTTTTTTTCTGCGC GTAATCTGCTGCTTGCAAACAAAAAAACCACCGCTACCAGCGGTGGT TTGTTTGCCGGATCAAGAGCTACCAACTCTTTTTCCGAAGGTAACTG GCTTCAGCAGAGCGCAGATACCAAATACTGTCCTTCTAGTGTAGCCG TAGTTAGGCCACCACTTCAAGAACTCTGTAGCACCGCCTACATACCT CGCTCTGCTAATCCTGTTACCAGTGGCTGCTGCCAGTGGCGATAAGT CGTGTCTTACCGGGTTGGACTCAAGACGATAGTTACCGGATAAGGCG CAGCGGTCGGGCTGAACGGGGGGTTCGTGCACACAGCCCAGCTTGG AGCGAACGACCACACCGAACTGAGATACCTACAGCGTGAGCTATGA GAAAGCGCCACGCTTCCCGAAGGGAGAAAGGCGGACAGGTATCCGG TAAGCGGCAGGGTCGGAACAGGAGAGCGCACGAGGGAGCTTCCAGG GGGAAACGCCTGGTATCTTTATAGTCCTGTCGGGTTTCGCCACCTCTG ACTTGAGCGTCGATTTTTGTGATGCTCGTCAGGGGGGC GGAGCCTATGGAAAAACGCCAGCAACGCGGCCTTTTTACGGTTCCTG GCCTTTTGCTGGCCTTTTGCTCA
**p15A**	TTTTTCCATAGGCTCCGCCCCCCTGACAAGCATCACGAAATCTGACG CTCAAATCAGTGGTGGCGAAACCCGACAGGACTATAAAGATACCAG GCGTTTCCCCCTGGCGGCTCCCTCGTGCGCTCTCCTGTTCCTGCCTTT CGGTTTACCGGTGTCATTCCGCTGTTATGGCCGCGTTTGTCTCATTCC ACGCCTGACACTCAGTTCCGGGTAGGAGTTCGCTCCAAGCTGGACTG TATGCACGAACCCCCCGTTCAGTCCGACCGCTGCGCCTTATCCGGTA ACTATCGTCTTGAGTCCAACCCGGAAAGACATGCAAAAGCACCACTG

	GCAGCAGCCACTGGTAATTGATTTAGAGGAGTTAGTCTTGAAGTCAT<br>GCGCCGGTTAAGGCTAAACTGAAAGGACAAGTTTTGGTGACTGCGCT<br>CCTCCAAGCCAGTTACCTCGGTTCAAAGAGTTGGTAGCTCAGAGAAC<br>CTTCGAAAAACCGCCCTGCAAGGCGGTTTTTCGTTTTCAGAGCAGA<br>GATTACGCGCAGACCAAAACGATCTCAAGA

Supplementary Sequences 2. Sequences of eight different TMS switches with different length of A and B sub-domains against the target GFP sequence 5'TTAAATTGCTAAGGAGATGAAATC3'

**TMS switch**	**sequence**
1	5'GGGCCCGGATAGCTCAGTCGGTAGAGCAGCGGAAAATAAGATCGAGTCGGATTTCATCTGCAA<br>AGCAATTTAACGACTCGAGAAGCCAGACCGCGGGTCCAGGGTTCAAGTCCCTGTTCGGGCGCCA3'
2	5'GGGCCCGGATAGCTCAGTCGGTAGAGCAGCGGAAAATAAGATCGAGTCGGATTTCACTGCAA<br>AGCAATTTACGACTCGAGAAGCCAGACCGCGGGTCCAGGGTTCAAGTCCCTGTTCGGGCGCCA3'
3	5'GGGCCCGGATAGCTCAGTCGGTAGAGCAGCGGAAAATAAGATCGAGTCGGATTTCCTGCAA<br>AGCAATTTCGACTCGAGAAGCCAGACCGCGGGTCCAGGGTTCAAGTCCCTGTTCGGGCGCCA3'
4	5'GGGCCCGGATAGCTCAGTCGGTAGAGCAGCGGAAAATAAGATCGAGTCGGATTTCTGCAA<br>AGCAATTCGACTCGAGAAGCCAGACCGCGGGTCCAGGGTTCAAGTCCCTGTTCGGGCGCCA3'
5	5'GGGCCCGGATAGCTCAGTCGGTAGAGCAGCGGAAAATAAGATCGAGTCGGATTCTGCAA<br>AGCAATCGACTCGAGAAGCCAGACCGCGGGTCCAGGGTTCAAGTCCCTGTTCGGGCGCCA3'
6	5'GGGCCCGGATAGCTCAGTCGGTAGAGCAGCGGAAAATAAGATCGAGTCGGATCTGCAAAGCAA<br>CGACTCGAGAAGCCAGACCGCGGGTCCAGGGTTCAAGTCCCTGTTCGGGCGCCA3'
7	5'GGGCCCGGATAGCTCAGTCGGTAGAGCAGCGGAAAATAAGATCGAGTCGGACTGCAAAGCA<br>CGACTCGAGAAGCCAGACCGCGGGTCCAGGGTTCAAGTCCCTGTTCGGGCGCCA3'

8	5'GGGCCCGGATAGCTCAGTCGGTAGAGCAGCGGAAAATAAGATCGAGTCGGCTGCAAAGC CGACTCGAGAAGCCAGACCGCGGGTCCAGGGTTCAAGTCCCTGTTCGGGCGCCA3'

Supplementary Sequences 3. Five sequences of the loop domain of the TMS switch and the reverse complementary sequence of the target RBS region in the GFP mRNA. These were tested with different stem lengths in Figure S3.

Sequence No	Sequence of the target region of the GFP	Sequence of the repressor domain of the TMS switch
1	5'GTTGGTTCCTAAGGAGCACTTTTG3'	5'CAAAAGTGGTGCATAGGAACCAAC3'
2	5'TTAAATTGCTAAGGAGATGAAATC 3'	5'GATTTCATCTGCAAAGCAATTTAA3'
3	5'TGCTGTAGGCAAGGAGATAGGCTT 3'	5'AAGCCTATCTCCCCGCCTACAGCA3'
4	5'ATTACGAAATAAGGAGAGCTTAGT 3'	5'ACTAAGCTCTGGGTATTTCGTAAT3'
5	5'AGTCAGAGTAAAGGAGGAATAGA A3'	5'TTCTATTCGACCATTACTCTGACT3'

Supplementary Sequence 4. Sequence of the construct to express pristine repressor RNA

**T7P-Repressor sequence-T7 TT**	5'TAATACGACTCACTATAGGGTTTTCACTTCCAATCCTTGGTTGCTAGC ATAACCCCTTGGGGCCTCTAAACGGGTCTTGAGGGGTTTTTTGGA3'

Supplementary Sequences 5. Sequences used in the orthogonality test, corresponding to Figure 1C.

Index No	Target region in the GFP mRNA	Repressor loop in TMS switch	Reverse complementary sequence in the loop of the anti-repressor
1	5'ATTGATTGT AAAGGAGGGC GTTTA3'	5'GCGGGACACATCGAGGCG TGTTAAACGCCAACCACTAC AATCAATACACGCCTACTTT AAGACCGC3'	5'GCGGTCTTAAAGTAGGCGTGT ATTGATTGTAGTGGTTGGCGTTT AACACGCCTCGATGTGTCCCGC3'

2	5'CTTCTGTGTA AAGGAGGTCG TTCT3'	5'GCGGGACACATCGAGGCG TGTAGAACGACTTCCTCTAC ACAGAAGACACGCCTACTTT AAGACCGC3'	5'GCGGTCTTAAAGTAGGCGTGT CTTCTGTGTAGAGGAAGTCGTTC TACACGCCTCGATGTGTCCCGC3'
3	5'TGTATTTGTA AAGGAGGTTC GTTT3'	5'GCGGGACACATCGAGGCG TGTTAAACGAAACCCAATA CAAATACAA CACGCCTACTTTAAGACCGC 3'	5'GCGGTCTTAAAGTAGGCGTGT TGTATTTGTATTGGGTTTCGTTTA ACACGCCTCGATGTGTCCCGC3'
4	5'TATTTGCTTG AAGGAGGTTC TTGG3'	5'GCGGGACACATCGAGGCG TGTCCAAGAACCAACACCA AGCAAATA ACACGCCTACTTTAAGACCG C3'	5'GCGGTCTTAAAGTAGGCGTGT TATTTGCTTGGTGTTGGTTCTTG GACACGCCTCGATGTGTCCCGC3 '
5	5'TATTCTTGTA AAGGAGGTCT TGTG3'	5'GCGGGACACATCGAGGCG TGTCACAAGACAGGGAGTA CAAGAATA ACACGCCTACTTTAAGACCG C3'	5'GCGGTCTTAAAGTAGGCGTGT TATTCTTGTACTCCCTGTCTTGTG ACACGCCTCGATGTGTCCCGC3'
6	5'TTTCTGTCTG AAGGAGGGTT ATTC3'	5'GCGGGACACATCGAGGCG TGTGAATAACCGTTTGGCAG ACAGAAAACACGCCTACTTT AAGACCGC3'	5'GCGGTCTTAAAGTAGGCGTGT TTTCTGTCTGCCAAACGGTTATT CACACGCCTCGATGTGTCCCGC3'

Colour code:

Sequence of the stem connecting the repressor domain to the tRNA

Sequence of the IBE

Sequence of the stem connecting IBE to the repressor

Sequences of the repressors

Sequence of the ribosome in GFP target region

Supplementary Sequences 6. RNA sequences to target FtsZ and T7 polymerase genes

FtsZ	5'CACAAATCGGAGAGAAACTATGTTTGAACC3'
T7 polymerase	5'TCCGGATTTACTAACTGGAAGAGGCACTAA3'

Supplementary Reference

1. J. Sambrook, E. F. Fritsch, T. Maniatis, Molecular Cloning: A Laboratory Manual, (Cold Spring Harbor Laboratory Press, Plainview, New York, edn. 2, 1989).
2. Lin-Chao S, Chen W-T, Wong T-T, *Mol. Microbiol.* **1992, 6,** 3385-3393.
3. G. Selzer, T. Som, T. Itoh, J. Tomizawa, *Cell* **1983,** 32, 119-129.
4. M. Zuker, *Nucleic Acids Res.* **2003,** 31, 3406-3415.
5. J. N. Zadeh, C. D. Steenberg, J. S. Bois, B. R. Wolfe, M. B. Pierce, A. R. Khan, R. M. Dirks, N. A. Pierce, *J. Comput. Chem*. **2011,** 32, 170–173.
